# How do we understand the value of drug checking as a component of harm reduction services? A qualitative exploration of client and provider perspectives

**DOI:** 10.1186/s12954-024-01014-w

**Published:** 2024-05-11

**Authors:** Lissa Moran, Jeff Ondocsin, Simon Outram, Daniel Ciccarone, Daniel Werb, Nicole Holm, Emily A. Arnold

**Affiliations:** 1 Center for AIDS Prevention Studies, Department of Medicine, University of California, San Francisco, CA 94143 USA; 2 Family & Community Medicine, Department of Medicine, University of California, San Francisco, CA 94143 USA; 3 Centre on Drug Policy Evaluation, St. Michael’s Hospital, Toronto, ON M5B 1W8 Canada; 4 Division of Infectious Diseases & Global Public Health, UC San Diego School of Medicine, University of California, San Diego, CA 92093 USA

**Keywords:** Drug checking, Harm reduction, Opioid, Fentanyl, Substance use, Overdose, Qualitative, North america

## Abstract

**Background:**

Mortality related to opioid overdose in the U.S. has risen sharply in the past decade. In California, opioid overdose death rates more than tripled from 2018 to 2021, and deaths from synthetic opioids such as fentanyl increased more than seven times in those three years alone. Heightened attention to this crisis has attracted funding and programming opportunities for prevention and harm reduction interventions. Drug checking services offer people who use drugs the opportunity to test the chemical content of their own supply, but are not widely used in North America. We report on qualitative data from providers and clients of harm reduction and drug checking services, to explore how these services are used, experienced, and considered.

**Methods:**

We conducted in-depth semi-structured key informant interviews across two samples of drug checking stakeholders: “clients” (individuals who use drugs and receive harm reduction services) and “providers” (subject matter experts and those providing clinical and harm reduction services to people who use drugs). Provider interviews were conducted via Zoom from June-November, 2022. Client interviews were conducted in person in San Francisco over a one-week period in November 2022. Data were analyzed following the tenets of thematic analysis.

**Results:**

We found that the value of drug checking includes but extends well beyond overdose prevention. Participants discussed ways that drug checking can fill a regulatory vacuum, serve as a tool of informal market regulation at the community level, and empower public health surveillance systems and clinical response. We present our findings within three key themes: (1) the role of drug checking in overdose prevention; (2) benefits to the overall agency, health, and wellbeing of people who use drugs; and (3) impacts of drug checking services at the community and systems levels.

**Conclusion:**

This study contributes to growing evidence of the effectiveness of drug checking services in mitigating risks associated with substance use, including overdose, through enabling people who use and sell drugs to test their own supply. It further contributes to discussions around the utility of drug checking and harm reduction, in order to inform legislation and funding allocation.

## Background

The opioid crisis in the U.S. consists of multiple overlapping and inter-related waves of surging opioid exposure, dependency, overdose, and death rates. Each wave has emerged from different eras of an evolving drug market and multiple intersecting contextual factors such as trends in pharmaceutical manufacturing and prescription, socioeconomic inequities, and positive supply shocks of both licit and illicit opioids [1–3]. Though its history can be traced back to the 1980s and 1990s, the past decade has redefined the crisis [4].

By the time the U.S. Department of Health and Human Services (HHS) declared the opioid crisis a public health emergency in 2017 [5], a wave of unprecedented magnitude had been on the rise for nearly 4 years, marked by the rapid proliferation of fentanyl and synthetic analogues into the drug market [4, 6]. Even as mortality from heroin and prescription opioids leveled off or decreased, opioid overdose and death rates rose precipitously [6]. From 2018 to 2021, the rates of opioid overdose deaths in the U.S. nearly doubled, and by 2021, roughly 9 out of every 10 opioid overdoses in the country (88%) were fentanyl-related [7].

### California

In California, home to the highest number of opioid-related deaths in the U.S. [8], the opioid overdose death rate curve from 2011 to 2021 tells a harrowing story. The third wave was later to arrive in California than the national average, but its onset was rapid and dramatic. Opioid overdose death rates more than tripled from 2018 to 2021, and synthetic opioid (e.g., fentanyl) deaths increased 7.2 times, responsible for 37% of opioid overdose deaths in 2018, and 86% just three years later [9].

In response, the California Department of Public Health has committed to the expansion and promotion of policies, programs, and services to combat the overdose epidemic, with a special focus on harm reduction and drug checking strategies [10].

### Drug checking services

Drug checking services (DCS) have garnered particular interest as an expansion of harm reduction strategies, as they offer the opportunity for people who use drugs to test the chemical content of their own supply [11, 12]. In doing so, people who use drugs may be afforded the possibility of changing their use behavior to remove or reduce the likelihood of harm [13, 14]. Multiple DCS have been operating in Europe for years—particularly in venues known for high rates of recreational drug use like music festivals [14, 15]—but are less common in North America. In the U.S. and Canada, DCS have emerged primarily in response to the needs of marginalized people who use opioids, and operate predominantly within the context of frontline services [16–18].

Though not mainstream or broadly implemented, studies from North America indicate that DCS are generally acceptable among people who use drugs [19, 20], and report that both service users and providers have expressed desire for better access to DCS, legal protections for those providing and using drug checking, and advanced technologies that provide information on drug concentrations—not just drugs present—at the point of care [21–24]. Several studies explore the potential impact of drug checking when used at various points along the supply chain [25, 26], with findings that suggest feasibility, acceptability, and uptake of DCS among drug sellers [27], noting particular importance to drug sellers who are embedded in their community and hold long-term trusted relationships with customers [28, 29].

Arguably the most common and well-known drug checking modality in North America are fentanyl testing strips (FTS), or lateral flow assays, which were originally designed for the clinical use of detecting fentanyl in urine samples, but have been publicly available for several years for modified use with drug samples [30–33]. FTS have been a powerful tool to combat accidental fentanyl exposure: they are small, portable, relatively accessible, and detect fentanyl in minute concentrations that could still be enough to trigger an overdose in an opiate-naïve individual [31, 34]. They have been found to be particularly useful for outreach and street use [13, 25, 35]. That said, FTS are not useful in the same way for those who intend to use fentanyl, where the overdose risk is not in the presence of fentanyl, but in the concentration and presence of additional adulterants like sedatives [36].

Drug checking technology has advanced, and continues to advance, such that a greater amount can be known about the chemical components of a drug sample in a shorter period of time, in a broader array of environments [37]. Multiple drug checking modalities can inform people who use drugs about the presence of unexpected adulterants, such as benzodiazepines and xylazine, among others. Technologies that offer the greatest specificity and sensitivity include Gas Chromatography Mass Spectrometry and High-Performance Liquid Chromatography, which can detect the presence and concentrations of a wide array of chemicals present in even small amounts in a sample, but must be used in a laboratory setting by a trained technician [37]. More flexible technologies have emerged, like Fourier-Transform Infrared Spectroscopy (FTIR) [38], which is semi-portable, and returns information on the main chemical components of a drug sample (above 5% concentration) in a matter of minutes [31]. Paper spray mass spectrometry is more expensive than FTIR but is just as fast, and provides quantitative results [39]. Today, multi-technology-based drug checking services are available in some areas as standalone programs, or as added components to existing harm reduction centers [30, 40].

These innovations continue to advance amidst complex and evolving social, legal, political, and funding conditions [11, 21, 41, 42]. Legally, drug checking can be complicated as a public service, requiring the handling and, often, exchange of illicit drug material, of which possession and distribution is often criminalized [21]. Harm reduction initiatives more broadly—DCS, syringe access services, naloxone distribution, HIV/HCV testing, wound care, supervised consumption sites, and medications for opioid use disorder (MOUD), among others—can at times be unpopular socially and politically, as stigma associated with addiction and drug use combined with concerns about the goals and practices of harm reduction can generate powerful community pushback [41–47]. Legislators and policymakers at local, state, and federal levels who rely on constituent support may therefore shy away from supporting various harm reduction strategies, despite endorsement from public health officials and robust evidence showing that harm reduction improves the health, survival, and recovery potential for people who use drugs, without compromising community safety [48, 49]. At the same time, California was one of several states to bring lawsuits against opioid manufacturers, distributors, and pharmacy chains, alleging that they played an active and/or negligent role in the genesis and exacerbation of the opioid crisis [50]. Of the $43.3 billion in settlement funds that have been awarded thus far, California may receive nearly $4 billion [51]. These funds are specifically earmarked for activities that are to include “prevention, intervention, harm reduction, treatment and recovery services.” [52].

As the opioid crisis reaches an unprecedented magnitude and strategies to address it are at once both a priority and a topic of controversy, we aimed to explore the value of drug checking services and their role within harm reduction more broadly. In this study, we report on qualitative data from providers and clients of harm reduction and drug checking services, to explore how these services are used, experienced, and considered. We aim to contribute to an existing qualitative evidence base exploring the value and utility of drug checking services, particularly as data are leveraged to inform political narratives, legislation, and funding allocation.

## Methods

For this study, we conducted in-depth semi-structured key informant interviews across two samples: a “provider” sample and a “client” sample. The “provider” sample consisted of individuals providing clinical and harm reduction services to people who use drugs, as well as drug checking subject matter experts such as researchers and program heads. The “client” sample consisted of individuals who use drugs and were receiving harm reduction services at an agency where multiple forms of drug checking were included in the services provided.

From June to November 2022, two authors (DC & LM) conducted in-depth semi-structured key informant interviews with 11 providers—8 working in the U.S., 2 working in Canada, and one working in both countries. Included in the sample were 2 clinical providers, 4 researchers, and 5 harm reduction service providers [Table 1].

**Table 1:** Provider participants by role and locus of work

	U.S.-based	Canada-based	U.S. / Canada
Clinicians	2		
Direct service providers	3	2	
Researchers	3		1

We employed purposive sampling of known providers first, then snowball sampling, contacting additional potential participants at informants’ recommendation. All potential participants were contacted via email and invited to participate. If the participant agreed, an appointment was made for the interview to take place over Zoom. Interviews lasted between approximately 45 and 60 min, and solicited provider perspectives on the state of the drug market in their area, the perceived needs of and challenges faced by their local client population, and their attitudes and experiences with drug checking methods and programs and integrating such programs into existing services. Verbal consent was collected at the outset of the interviews, which were then recorded. Audio from the recordings was isolated and transcribed using a secure third-party professional transcription service. All transcripts were deidentified and researchers created unique anonymous ID numbers for each participant. Participating providers were offered an honorarium of $100 in the form of a gift card. The study protocol was reviewed by the University of California San Francisco Institutional Review Board (IRB #22-36262).

Client participant (*n* = 13) recruitment and data collection took place over a one-week period in November 2022 [Table 2].

**Table 2:** Client participant demographics

	Male (*n* = 8)	Female (*n* = 5)	Nonbinary (*n* = 0)	Total (*n* = 13)
Race				
Black	2			2
Latinx		1		1
Nat. American	1			1
White	5	4		9
Age				
18–29		1		1
30–49	7	4		11
50–64				
65+	1			1

We employed a non-random convenience sample, recruiting from four harm reduction programs in San Francisco, where clients were approached either by interviewers (NH & JO) or program staff who had been instructed on eligibility requirements. Eligible participants were at least 18 years of age, and currently using fentanyl, heroin, or methamphetamine. Clients were excluded from eligibility if they were intoxicated or otherwise unable to provide informed consent. Given that current drug use was an eligibility requirement, we assessed “intoxicated” as an inability to respond to simple questions, providing responses that are incoherent or unintelligible, or if the participant indicates that they are too high to continue. Potential participants who were eligible and interested were then formally verbally consented and interviewed on-site. Client interviews explored participants’ history of drug use and experiences with harm reduction services, as well as their awareness of, attitudes about, and experiences with various drug checking modalities. Interviews lasted approximately 30–60 min and were recorded, then submitted to the same external third-party transcription service being used for provider interviews. Participants were provided a $25 cash incentive as a token of appreciation for their time and expertise, and were provided unique ID numbers to anonymize their data. This study protocol, distinct from the protocol covering provider interviews, was reviewed and approved as well by the UCSF IRB (#22-36640).

### Analysis

Client interview transcripts were uploaded to Dedoose, a qualitative analytic program [53]. Four analysts (EA, LM, SO, and JO), two of whom were involved in data collection (LM & JO), read transcribed interviews from both client and provider data sets and drafted summaries which were then systematically reviewed as a team. Following the tenets of thematic analysis and adopting the framework developed by Miles and Huberman (1994) [54], the team collaboratively identified cross-cutting themes from interview summaries, covering areas of concordance, discordance, and particular importance, as well as exemplar and negative cases. Once major themes and sub-themes were identified and articulated, authors drafted analytic memos which consolidated and explored in detail each major theme.

Following publication of an article focused on findings from the provider data set [55], further analysis of the client data set included the development of a formal coding scheme (SO), based on *a priori* codes extracted from the interview guide, as well as codes reflecting themes and sub-themes identified in the summarizing process and further refined via ongoing weekly analytic meetings. Coding was led by the primary qualitative analyst [SO] with secondary coding by client interviewer and author [JO]. The application of codes was discussed regularly among all team members, focusing on discrepancies between primary and secondary coders, insights developed, and the potential emergent themes. Discrepancies occurred approximately 10% of the time, and these were resolved through group consensus in accordance with established qualitative research methods [56].

## Results

Through key informant interviews, we captured diverse perspectives on how existing and emerging drug checking services are being used, and their potential for future impact within the harm reduction suite of services.

We present our findings within three key themes: (1) the role of drug checking in overdose prevention; (2) benefits to the overall agency, health, and wellbeing of people who use drugs; and (3) impacts of drug checking services at the community and systems levels.

### The role of drug checking in overdose prevention

Service providers and clients expressed varying opinions on the extent to which information from drug checking services would prevent overdose and, indeed, whether overdose prevention is the appropriate metric by which drug checking’s impact should be measured. Clients reported diverse experiences and perspectives on how they use (or don’t use) drug checking, and expectations for their own future use.

### Fentanyl test strips

Almost all client participants reported having had some experience with fentanyl testing strips (FTS), either using them personally or seeing others use them. Attitudes about FTS varied. Some expressed concern that they are difficult to use correctly or that they have heard they may be unreliable (prone to false positives or negatives):

*We were using them constantly when they were telling us that all the drugs had fentanyl in them. But then we found out that if you don’t put enough water on speed, that it can come up positive because of some chemical.* [Client, 40, female].

Others reported relying on them heavily and using them often:

*I’ve just got to have that insurance that there’s no fentanyl in [my drugs]. … I have a drawer. Like that? That’s all full of test strips. Usually every time I come to a needle exchange, if they have them, I grab as many as I can and just put them in the drawer.* [Client, 43, male].

### Spectrometry

Although many had not heard of spectrometry, spectroscopy, or anything beyond FTS, once it was described what a range of drug checking services could look like, clients were interested and excited about the possibilities. Some expressed interest in using mobile or site-based spectroscopy, but were concerned about their safety, one expressing worry about “*judgment from the community*” or bystanders taking videos and calling the police, another wondering if they would be an “*easy target*” for law enforcement harassment. Those who reported having used FTIR as part of their harm reduction visits, however, had positive things to say:

*Interviewer: And how do you feel about that testing service at the van?*

*Participant: I think it’s remarkably great.*

*Interviewer: yeah?*

*Participant: Yeah. They answered my questions, exactly what I wanted to know. *[Client, 66, male]

Some participants described high percentages of testing experiences coming back with a positive or unexpected result, like a client who said that he’d used the FTIR mobile service four times with meth from four different suppliers, and “*only one came back pure*.”

### Using drug checking results

What participants reported doing with the results of checking their drugs varied as well. Some participants spoke about specific situations where drug checking prompted them to avoid buying contaminated drugs.

*Actually I just used [drug checking] yesterday. Luckily, I didn’t buy the heroin I was going to, because it tested for fentanyl*. [Client, 32, male]

Other community members expressed disinterest in checking drugs, often citing a lack of realistic options for using test results in a way that made sense for them. One participant stated directly that they didn’t want to test because they didn’t want to have to not use drugs if they got a result they didn’t like:

*What if it comes up with fentanyl in it? Then I bought it but I can’t do it? They’re not going to take it back, the people I bought it from. I mean even if I get them to write me a receipt, you know?* [Client, 49, male]

Another client said that she was interested in drug checking generally, but wouldn’t bother if she only had a little bit and was relying on it to keep her from getting sick:

*If I was trying to [check my drugs], I would do it when I had enough to do that, you know. Because if I was dope sick and I only had two hits of fentanyl, I probably would not [test].* [Client, 24, female]

Data from service provider interviews echoed these dynamics. We heard from provider participants that, broadly, drug checking services prevent overdose directly some of the time, but not all the time, by way of individual behavior change on a case-by-case basis. One provider—a clinician with a lengthy career in addiction medicine and harm reduction—echoed doubts about how common it would be for a patient to make use choices based on drug checking results, broadening the focus to personal harm reduction behavior change rather than abstinence behavior alone:

*And then the question is, what do you do about it? I’ve had a patient who is, like, yeah, I tested it. It was positive for fentanyl. I go, well, what did you do? Well, we just used anyway because it’s all we had. And we had, like, the Narcan out, and I – I just felt really sleepy afterwards. … So I guess that’s the other question – if you do drug testing and it isn’t what you expect, like, you can’t take it back to the dealer and say, hey, this isn’t – I want a refund; right? So what do you do with that information? And if, you know, if you’re in withdrawal and you really need to use that drug, like, what kind of safeguards are you going to take if you decide, yeah, I’m going to go ahead and use this; right?* [Clinician, U.S.]

Other service providers similarly drew a distinction between drug checking sparking behavior change that *prevents* overdose versus behavior change that *reduces the risk of death* from overdose, situating drug checking services as a set of tools that dovetail with existing personal harm reduction strategies.

*The reality is, you know, people still are using their drugs. Now, a large proportion of people who use our service say that they’ll do something differently after, you know, accessing our service, so they maybe will do a test dose first, or start, like, start with a smaller dose, or use with a friend, or use at an SCS [supervised consumption site].* [Direct service provider, Canada].

### Overdose prevention versus overdose rates

Interestingly, many service providers when asked for their perspective on the role of drug checking services in overdose prevention expressed concern about a gulf between the overdose prevention they observe at the service level versus what they see represented in population-level data.

*Will drug checking save a life? Absolutely. Yes, for sure. Will it, at a population level, drop overdose rates? I don’t know the answer to that.* [Researcher, U.S.]

Participants offered multiple explanations for this. One described challenges inherent in proving prevention, while another explained how population overdose rates can obscure the impact of drug checking programs when they operate within a rapidly-changing drug supply:

*It will be very hard to prove within these prevention paradoxes. I think prevention is one of those things that is so important, but within our scientific frameworks … preventable events are so rare and on the grand scheme of things, they’re really hard to prove. … But will [DCS] save lives? Yeah.* [Clinician, U.S.]

*The numbers aren’t showing [an overall decrease in overdose], right, because at the same time, even though we’re offering this service, the supply is just getting worse and worse, so overdose rates are rising.* [Direct service provider, Canada].

Not every participant who commented on this gulf found it to be wide or troubling, but instead remarked on it as a neutral distance between two related but distinct constructs, one of which is a measure of what outcomes drug checking information could yield, and the other of which is a fundamental right to that information.

*It’s really a great question if we’re going to see things pan out in the numbers. I certainly hope so and I certainly think so, but I think that we just have the right to know what we’re putting into our bodies, regardless of what outcome measures are. We deserve to know what’s in our drugs*. [Direct service provider, U.S.]

Similarly, a direct service provider offered a structural perspective on overdose prevention, decoupling the value of drug checking services from overdose outcomes, prioritizing instead the intrinsic value of equipping people with critical information about what they are putting in their body and the importance of empowering people to make decisions with as much information as possible.

*I don’t really know if [drug checking] is going to decrease the rate of overdose. In my mind, the problems that contribute to overdose are prohibition, law enforcement harassment, and everything that surrounds that that creates a shitty drug supply and then prevents people from investigating it.*

*But what [drug checking] does do, again, is this piece around like, people should know that they can find out there’s more in their drug. … I think that it just enables people to make better educated decisions around their substance use and to understand their bodies better*. [Direct service provider, U.S.]

### Benefits to the overall agency, health, and wellbeing of people who use drugs

Drug checking services offer users the tools to independently identify risks in the drug supply and make decisions about what to do with that information in the short and long term. Many of the service providers interviewed for this study, when asked how drug checking would impact overdose rates, gave some version of a reframed response, repositioning the focus from the drug use decisions themselves to the importance of information in fortifying the overall agency, health, and wellbeing of people who use drugs.

The provider quoted in the above section went on to reflect on the intrinsic value of giving people information, arguing that it contributes to essential experiences of bodily autonomy and health equity:

*What’s really important to me as well is just sort of building this momentum around people feeling entitled to bodily autonomy and seeing that [drug checking] is a part of [that], and having folks know that, yeah, they fucking deserve to have this information. They are entitled to know what is in their stuff. And so, that’s not the* only *piece to health equity and justice around substances and substance use, but I think that it’s a significant piece.* [Direct service provider, U.S.]

Knowledge of what is in their drugs can also confirm users’ internal experience. One provider, who had piloted an early drug checking intervention in a major metropolitan area in the U.S., believed that drug checking for people who use drugs offers confirmation of the embodied experience of their substance use, which in this provider’s experience was often regarded with skepticism by health workers:

*I think that people are able to connect experiences that they’re feeling in their body with real information. And I think that actually validates the really organic knowledge and experiential knowledge of drug users as the true experts about drugs. You know, when we were doing our project in [city] and fentanyl was not everywhere [yet]—almost 100% of the time, if someone brought us a sample and said, “I think this has fentanyl in it,” it was true. … It validates experience where people’s experiential knowledge is not really validated by an educational system. It’s always this kind of thing where public health people are telling drug users what’s true. And drug checking sort of validates that drug users actually know what’s true, and we’re just using science to confirm it.”* [Direct service provider, U.S.]

Client interviews echoed this theme. Several clients recounted experiences that illustrated how navigating the drug market is becoming increasingly difficult, and that drug checking provides an important tool that they can pair with their own instincts and expertise as they try to keep themselves safe.

*I can look at it and I can be like, “Wait a minute, we might want to test that.” Because speed and fentanyl are different. They actually look different than the other one, so when I start seeing traces of fentanyl being in the speed, I go, “We need to check that before we do any of it.” And, hey, sometimes I’m wrong.* [Client, 43, male]

*The [meth] that was in the medicine bottle [tested positive for fentanyl], yeah. But I kind of knew it was going to because I packed a bowl right before and if it’s dirty … yeah, the color starts changing wrong right away.* [Client, 43, male]

*I like that [drug checking] gives us some certainty of what’s in the drug … like with the heroin, there was stuff in that that just did not feel good. I’d love to know what they were cutting that stuff with. We used to joke it was shoe polish because it was so dark and dirty, but it’s really important what you put in your body*. [Client, 48, female]

Our client data further provide evidence that people who use drugs are making health-related decisions for themselves and care about their own health and wellbeing. Woven throughout community member interviews were examples of health-seeking decision-making in users’ everyday lives, demonstrating agency in considering health behaviors and expressing both implicitly and explicitly a desire to care for themselves. Examples of these pro-health micro-decisions include choosing not to smoke out of foil (it’s “*not healthy to smoke out of*” and “*it’s going to give us Alzheimer’s or something*”) or reducing smoking marijuana due to a “*sensitive*” respiratory system. One informant laid out explicitly their hopes for their future, shaped too by an acute awareness of the risks of the current drug market:

*I don’t want to be a statistic out here. I want to go back to regular life and experience all the rest of the highs that there still are out there before I die. I want to jump out of an airplane, or take a balloon ride, or ride more rollercoasters. … I don’t want to limit myself to one freaking high. … it’s not worth it anymore at all. … You’d never OD on meth before. Meth and weed were two things you just didn’t overdose on. If you did too much, you passed out and you slept it off and that was it. Now, no matter what drugs you’re doing, every time you use, it’s a 50–50 chance that you could die.* [Client, 49, female]

These excerpts from client interviews highlight the demand among potential DCS users for strategies that contribute to their agency, health, and wellbeing, even within the context of continued drug use in the short- or long-term.

### Impacts of drug checking services at community and systems levels

In addition to use at the individual level, participants talked extensively about the ways that they experience and imagine DCS having an impact at community and systems levels. They described the ways that drug checking could facilitate upstream regulation of the drug market, how the information and transparency made possible by checking drugs can fill a policy and regulatory vacuum, and how drug checking can empower public health surveillance systems and clinical response.

#### Community level regulation of the drug market

Multiple informants, both service providers and clients, reflected on the use—or potential use—of drug checking as a grassroots tool to regulate the drug market.

Participants talked about using, or thinking one could use, DCS as a vetting tool for sellers or suppliers.

*And if people could get their shit tested, almost every time if not every time, not only would it help them to be safer by them regulating themselves and knowing what’s in their stuff … But I feel like if they knew exactly what was in it, they could go tell their guys that they got it from, “Look, man, I’m not buying that shit anymore if it’s like that. If that shit -- if this or that’s in it or whatever. Or if you don’t, whatever, I’m not buying it from you. I’m buying it from someone else.” And that might even make them be… It’ll hold them more accountable.* [Client, 32, male]

This use was so important to one participant that they expressed interest in their samples being sent for more extensive in-lab spectrometry testing that could give them greater detail about the compounds and amounts in their sample:

*Hey, [a full spectrometry report] may take a week, but at least in that week, I find out if I should go back to that person or not.* [Client, 43, male]

Client participants frequently referred to DCS as a tool to “keep [suppliers] honest”; that is, as informal regulatory pressure on currently unregulated illegal drug markets. Some reported that they spread the word if drugs from a supplier come up contaminated or low-grade. One participant, who uses fentanyl, reported using FTS to ensure that what they are about to buy is, indeed, fentanyl:

*I keep them [FTS] around. … Then I say, “Can I test it?” and I test it in front of them. And like some of it’s turned up negative. And so I totally outed them out on the block with it. It pisses them off – it kind of keeps them honest.*

*… When you got a bunch of test strips, I can go down the line and keep, yeah, at least trying to keep them honest, you know. I got a pile of those things right now. That’s actually what I use them for.* [Client, 40, male]

Of particular value, according to our participants, was the idea that spectrometry would provide formal documentation of drugs’ contents. Analytical evidence that something was either dangerously contaminated or not what the seller claimed it to be can shift the balance of power in the transactional dynamic, placing upstream pressure on suppliers to better monitor what they are contributing to the market.

*If you could get results that are on paper or on a text or on a whatever, then you could bring it to them that, “Look, dude. I’m not fucking around. You need to make this shit right or I’m not buying it anymore.” That would be a game-changer*. [Client, 32, male]

From the service provider standpoint, one participant, a drug checking technician and program manager with a longstanding history in their city’s drug scene, identified similar opportunities for DCS to impact the drug market, were it made easily accessible to those at multiple points in the drug supply chain in addition to consumers.

*It’s not just people who are consuming the drugs that can use the service. It’s also people who are selling them. And so, oftentimes people who are not essentially the first or second hands that are creating the substance and then moving it down the chain towards the end consumer, they don’t know what is in their product. For folks who are selling drugs, if they’re able to come and get an ingredient list, they can then kind of know what to say to folks who are buying.* [Direct service provider, U.S.]

This was not discussed as just a hypothetical. One informant who sells drugs validated this use as feasible and valuable:

*I want to make sure what I’m buying is what it is. … I do sell it myself, so [spectrometry]’s a good service because that’s what I want to know is the chemical balance as to how much it is and how much it isn’t and whether it’s good every time.* [Client, 66, male]

#### Filling a policy and regulatory vacuum

In the absence of a government or regulatory body that will monitor and report on the verified contents of illicit drugs, our data suggest that drug checking services, and spectrometry in particular, may be filling a policy and regulatory vacuum.

Clients likened the idea of having access to a list of drugs present in a sample to knowing ingredients of something that they would eat.

*I mean we know what’s in our food, right? The packaging is all labeled and the ingredients are listed. It’s just too important, especially with drugs. Especially because we don’t know who’s making them. We don’t know exactly where they’re coming from. And every single one is different. Every week is different. Even if you buy it from the same person all the time, they’re always having something different. Maybe you’ll have the same thing twice or three times but that’s it.* [Client, 48, female]

Providers, meanwhile, explicitly framed the value of drug checking within the context of an unmet regulatory need. One service provider qualified many of their statements about drug checking services with “until prohibition goes away,” situating DCS as being necessary only in a regulatory vacuum. Another spoke more directly to the relationship between drug checking and regulation:

*And with drugs, because of prohibition, we just have this unknown, unregulated supply, and people are – what they’re putting in their bodies and what they’re purchasing is obscured, right? And so, drug checking is like a series of sort of imperfect tools to help consumers of drugs regain a little bit of control in the form of information around what it is that they are using. …. And there’s a very good argument that, if we had some kind of safe, regulated supply, we wouldn’t need drug checking at all, which is true*. [Direct service provider, U.S.]

#### Empowering public health surveillance systems and clinical response

Data from our interviews suggest that drug checking technologies and programming may also contribute meaningfully at a structural level, to public health surveillance systems and clinical response. Aggregated sample results provide real-time data about what drug compositions are trending across regions, and what the clinical implications may be for providers treating clients who use drugs [57]. One drug checking program team posted results to their website in the hopes of informing local clinicians and public health policy makers about what was circulating in the drug supply. This program manager talked about making results available “at the societal level”:

*And then at the kind of societal level what we do … [is] every other week we take all of the results from the samples that we’ve checked, and we combine them, and then we put out a report and update our website about, like, what’s circulating in the drug supply. So we talk about, you know, trends in the drug supply over that period, and new drugs that have been introduced, and what those drugs could mean, that type of thing. So service doesn’t only benefit individuals, but it also benefits the larger community by being able to say, okay, this is what we’re seeing. If you can’t access the service, you still at least know, you know, what is circulating.* [Direct service provider, Canada]

Community members expressed an awareness of this function. One participant cited drug checking’s role in a larger tracking network as one of the things they value most about the service:

*I liked a lot about [drug checking]. One, that it was available in the first place. Two, that it was not just doing its own thing. It was part of a larger network that was keeping track of what drugs were popping up on the streets and what their makeup was. I really like that that’s happening.* [Client, 30, male]

At the point-of-service level, provider informants discussed significant benefits that drug checking could provide to clinicians and other medical professionals who work closely with people who use drugs. This informant posited specifically that having more detailed knowledge about what was circulating in the drug supply could help clinicians better formulate strategies for managing opioid use disorder and transitioning patients onto MOUD:

*Understanding what’s actually in the supply… allows clinicians to tailor the care that they are providing to people who use drugs. So, you know, if they know that the average amount of fentanyl in a fentanyl sample is this and they want to transition someone off the unregulated drug supply onto, like, a pharmaceutical alternative, well, what pharmaceutical alternative is actually suitable based on what they’ve been using?* [Direct service provider, Canada]

This is especially critical given the significant difficulties that have been recently reported when transitioning people using fentanyl to appropriate longitudinal services [58]. A provider we interviewed who runs a mail-based drug checking service in the U.S. reported that developing a more thorough knowledge of the drug supply outside of the current surveillance panoply may provide important clinical toxicology assistance to help physicians connect health outcomes to specific substances or components of the drug supply, and more quickly provide tailored treatment:

*There’s one other really big one for me, which is that it allows us to link specific physiological harms with specific chemicals. So, we’re not just talking about dope anymore. We’re talking about this component of dope causing this specific reaction. What we have been able to do is, we’ll get calls from our central hospital on campus, and they’ll say, “We have this patient with an idiosyncratic presentation. Boom, boom, boom, boom, boom, boom. Here it is. We think it might be… You know, they’ve been injecting this, this, and this. We have some of their samples. Can we get them tested?”*

*Or if they don’t have the samples, they’re like, “This is what the symptoms are. This is where they’re from. What are you seeing about the drug supply in their area?” And I can be like, “Well, yeah, there’s been a spike in levamisole in that area or xylazine,” you know, whatever it is. And then they can get to treatment quicker because the physicians have a more specific knowledge about the ideology of the harm that they’re observing in clinic.* [Researcher, U.S.]

### Negative cases

While the vast majority of participant responses reflected positive experiences with or attitudes about DCS, some participants additionally expressed ambivalence or concern. Many of these perspectives are embedded within the themes reported above, but deserve reiteration: service users expressed concerns about the accuracy of drug checking technologies, their privacy and safety relative to community stigma and law enforcement, and anxiety about having to make hard choices about drug use in the face of an unexpected result. Service providers expressed concern about the “then what” of drug checking, citing constrained choices and limits to what could be realistically expected in terms of behavior change without other supports in place. Some further lamented the challenges of translating the benefits of what they were seeing in practice to what is visible to a broader audience.

Not included in the above findings, but important to note, are two additional concerns that arose in interviews. First, service users and providers cautioned that the street drug supply changes so quickly that new compounds may be showing up on the street before they are identified in spectrometry libraries, potentially limiting their ability to accurately identify contaminants. Finally, one provider, a clinician with a longstanding career in addiction medicine and harm reduction, closed their interview with a somber caution against decontextualizing drug checking from a broader commitment to multi-method harm reduction, health equity, and social justice.

*[I worry that] we’re just throwing yet another technology at a much bigger problem. My fear is that people will say, oh, now we have drug checking, so now we can stop trying to dismantle, you know, structures of racism and oppression in society, right? We can stop looking for homes for people because we have this technology that’s going to prevent people from dying. … It doesn’t work that way.* [Clinician, U.S.]

## Discussion

While the magnitude of the opioid crisis is often communicated in terms of overdose and death rates, the harms associated with opioid use—intentional or unintentional—in an unregulated drug market extend far beyond those data points alone, and so too must the strategies leveed to combat them. Our findings demonstrate that drug checking services offer diverse benefits at the individual, community, public health, and health systems levels.

### Overdose prevention and beyond

If the question is, *do and will these technologies contribute to overdose prevention*, our findings suggest that the answer is yes, with some important caveats. The first being that, according to our participants, they do not prevent overdose all the time. Our findings reflect that individuals make complex and highly contextualized decisions regarding their use behavior each time they use drugs. Information about the chemical composition of a drug sample sometimes leads to decisions to abstain, but more often leads to decisions to engage in other types of harm reduction behaviors—like using with a friend rather than alone, making sure to have naloxone on hand, using at a supervised consumption site, alerting others to a bad batch, using a tester first, or avoiding a certain supplier in the future. Sometimes it leads to no observable behavior change at all.

Further, DCS have not been scaled up to meet the needs of everyone at risk for overdose; until it is, it is premature to discuss population-level prevention. This study does not purport DCS to be in and of themselves sufficient to prevent overdose, but they are clearly part of a continuum of services that can prevent overdose mortality.

Many participants took care to note as well that the needs of people who use drugs are not solely to avoid overdose; people navigating drug use are whole people, and the stigmatization and criminalization of drug use regulates their access to a multitude of essential needs and liberties, like health care, housing, employment, agency, and a host of social and legal protections. Access to information that contributes to agency and autonomy, and enables more informed decision-making, is an essential service regardless of other outcomes.

Of course, among harm reductionists and researchers acquainted with the diverse and dynamic ways that harm reduction functions within communities, this is not news. Our findings reflect and reinforce much of the existing evidence from studies aiming to understand the role of drug checking within the larger constellation of harm reduction and, indeed, the role of harm reduction itself.

One recent qualitative study in particular reported themes with striking similarities to the prevailing themes from our interviews. Wallace et al. [59] explored the potential impacts of community drug checking on prospective service users, finding drug checking to “increase quality control in an unregulated market,” “improve the health and wellbeing of people who use substances,” and “mediate policies around substance use.”

Our findings further add to existing evidence that links drug checking with consumer empowerment within an opaque drug market [25, 26, 29] and underlines the reciprocal relationship between individual agency and the adoption of harm reduction strategies [46, 60, 61].

Of note is the shifting context in which many existing drug checking studies, including ours, are situated. In some areas, fentanyl appears most often as an unwanted adulterant in another drug—be it a non-opioid or a less potent opioid like heroin—and DCS are used primarily for fentanyl avoidance [13, 19]. Increasingly, however, pockets of consumers are preferring fentanyl, as seen in our San Francisco client sample and within populations reflected in recent drug checking studies. Our data echo the broader finding that drug checking technologies are likely to be used differently by fentanyl-seeking opioid users versus fentanyl-avoiding opioid users, and differently still among those using stimulants, psychedelics, or other non-opioid drugs [22, 62].

On the subject of behavior change—whether and how drug checking can be understood to prompt changes in drug use behavior—our findings align with existing evidence showing that drug checking is at times followed by contaminated drug disposal, and at times followed by the employment of personal harm reduction techniques such as spreading information within the community [30, 63], and reduction in polysubstance use or dosage [13–15, 64]. Lacking as we do a robust methodological-empirical foundation to assess this type of causality, whether and to what extent drug checking in various contexts leads to less use or more safe use among different populations cannot be stated concretely [16, 65, 66]. Whether individuals change their use behavior based on drug checking results is highly informed by such matters as how limited their access to drugs is, realistic options for modified use, and their perceived relative risks of knowingly ingesting a potentially dangerous compound or compounds versus not.

### The tension at the center of harm reduction policy

The role of harm reduction services within communities have long reflected a central tension: in contrast with abstinence and criminalization models, harm reduction is often socially and politically criticized as enabling drug use and making neighborhoods less safe [67–69], while research consistently finds harm reduction to yield positive outcomes for both service users and surrounding communities [70, 71]. In addition to improving the health and wellbeing of people using drugs, evidence suggests that those accessing harm reduction services are more likely to ultimately seek treatment and pursue recovery [49, 70, 72, 73]. Concerns about public safety, too, while in many cases expressed in good faith, have been shown to be largely misplaced: multiple studies show harm reduction programs to have no significant impact on nearby violent or property-related crime, with some findings suggesting improved indicators of public order and safety [48, 49, 74, 75]. Harm reduction strategies have additionally been found to be cost-effective in the short term and cost-saving to public monies in the medium- and long-term [76]. Nonetheless, public perception of harm reduction has historically been interwoven with deeply entrenched cultural stigmas related to race and ethnicity, socioeconomics, and an imprecise moralism that positions access to health and protection as a privilege that should be earned or denied based on behavior [67, 69, 71].

This tension plays out most concretely in the public policy space. Even as the opioid crisis dominates public health discourse and funding is earmarked for research and programming to combat it [77], harm reduction programs on the ground are under siege. At the federal level, the House Appropriations bill for the Fiscal Year 2024 HHS budget dramatically cuts funding to HIV/AIDS programs—a budget umbrella under which many harm reduction, substance use support and treatment programs are funded [78, 79]. In California, a $15.2 million state grant supporting syringe access services has dried up amidst an overdose crisis at its peak, with no plans for replacement [80]. In 2022, a landmark bill (SB58) that would have authorized overdose prevention programs with supervised consumption in Los Angeles, Oakland, and San Francisco was vetoed by the Governor, despite broad support and robust evidence behind it [81]. Funds for such safe consumption sites have further been excluded from receiving opioid settlement funds in San Francisco [82], and in September of 2023, a bill was put forth by the San Francisco Mayor’s office to require drug screening and mandatory treatment for anyone receiving public services [83]. This, despite the expressly articulated commitment to and acknowledged necessity of harm reduction services—services explicitly aimed at helping people who use drugs to be more safe rather than abstaining from use—highlighted in policy language across multiple levels of government and legislature [10, 84–87].

It is worth noting that one of the harm reduction sites where several of this study’s client participants were receiving services was defunded shortly after we completed data collection, and since then, overdose death rates in the city have climbed [88] and public order in that area has reportedly deteriorated [89].

### The framing of effectiveness is crucial in this policy environment

In light of these tensions, we offer the findings of this study as a contribution to an evidence base that may play an increasingly central role in California’s—and the nation’s—opioid crisis response. The allowable expenditures for opioid settlement funds list “evidence-informed programs to reduce the harms associated with intravenous drug use” as a focus area [51] and California’s Overdose Prevention Initiative describes its approach as being “data-driven.” [10] The proposed HHS FY2024 budget, in addition to cutting much of the funding that covers harm reduction programming, proposes the rejection of “controversial programs” while maintaining funding for “an effective opioid response.” [78] As California faces a $68 billion budget deficit [90] and supplementary federal and settlement funds are to be apportioned based on strategy effectiveness and the body of scientific evidence, the role of research comes into sharper focus. It is the strength or weakness of the evidence base—of the complexity of the research inquiry and integrity of the data—that may ultimately frame which initiatives are eligible for support.

When asked about the place and promise of drug checking within the broader constellation of harm reduction services, it was drug users’ humanity and right to health, more so than the public health implications, that grounded many of our participants’ responses. Their responses implicated, too, the underlying operating principle that, ultimately, people make choices that make sense for them. Whether by the hand of addiction or desire, constrained options or access, or every individual’s complex hierarchy of relative dangers and needs, people’s choices are reflections of their full humanity. Approaches to stemming the tide of this crisis cannot be effective unless they are built on respect for the individuals living it, and focused on understanding their needs.

We encourage continued research and reporting on drug checking services and emerging technologies, with an emphasis on exploring effectiveness within a broad scope, reflective of the impacts of these services on whole lives and systems.

### Limitations

Many of the community members we interviewed had not heard of spectrometry or spectroscopy, and the interview represented the first time they were introduced to the technology as a concept and the first time they considered whether and how they could see themselves using it in their own lives. This limits the range of our findings among the client sample, given that much of our qualitative data speaks to hypothetical future use rather than past or current use of emerging technologies. The absence of data on client use should not be interpreted to mean that participants chose not to use DCS.

Additionally, the sampling frame for clients was limited to one setting, while providers were sampled from across North America, and the small sample size for both groups may have limited saturation. Finally, providers did not reflect all North American regions where drug checking has been implemented, nor all DCS models, limiting the generalizability of findings.

## Conclusion

Our manuscript contributes to growing evidence of the effectiveness of drug checking services in mitigating a range of risks associated with substance use, including overdose, and offer diverse benefits at the individual, community, public health, and health systems levels. For that reason, policymakers should consider allocating resources towards its implementation and scale-up in settings impacted by overdose mortality.

## Data Availability

Due to ethical restrictions, the data generated and analyzed during the current study are not available to those outside the study team. Data and materials are of a sensitive nature, and participants did not consent to transcripts of their interviews being publicly available. Portions of interviews about which editors have questions or concerns may be provided upon request after any details that may risk the confidentiality of the participants beyond de-identification have been removed. Researchers who meet the criteria for access to confidential data may send requests for the interview transcripts to the Human Research Protection Program (HRPP)/IRB at the University of California, San Francisco at 415-476-1814 or IRB@ucsf.edu.

## Abbreviations

DCS: Drug checking services
FTIR: Fourier–Transform Infrared Spectroscopy
FTS: Fentanyl testing strips
HHS: US Department of Health and Human Services
MOUD: Medications for opioid use disorder
